# Inhibitors of apoptosis proteins (IAPs) expression and their prognostic significance in hepatocellular carcinoma

**DOI:** 10.1186/1471-2407-9-125

**Published:** 2009-04-27

**Authors:** Claudia Augello, Luca Caruso, Marco Maggioni, Matteo Donadon, Marco Montorsi, Roberto Santambrogio, Guido Torzilli, Valentina Vaira, Caterina Pellegrini, Massimo Roncalli, Guido Coggi, Silvano Bosari

**Affiliations:** 1 Department of Medicine, Surgery and Dentistry, Division of Pathology, University of Milan, AO S Paolo e Fondazione IRCCS Ospedale Maggiore Policlinico, Regina Elena e Mangiagalli, Milan, Italy; 2 Department of Pathology, University of Milan, IRCCS Istituto Clinico Humanitas, Rozzano, Milan, Italy; 3 3rd Division of General Surgery, University of Milan School of Medicine, Istituto Clinico Humanitas IRCCS Rozzano, Milan, Italy; 4 Division of Bilio-Pancreatic Surgery, AO S Paolo, Milan, Italy

## Abstract

**Background:**

Similarly to other tumor types, an imbalance between unrestrained cell proliferation and impaired apoptosis appears to be a major unfavorable feature of hepatocellular carcinoma (HCC). The members of IAP family are key regulators of apoptosis, cytokinesis and signal transduction. IAP survival action is antagonized by specific binding of Smac/DIABLO and XAF1. This study aimed to investigate the gene and protein expression pattern of IAP family members and their antagonists in a series of human HCCs and to assess their clinical significance.

**Methods:**

Relative quantification of IAPs and their antagonist genes was assessed by quantitative Real Time RT-PCR (qPCR) in 80 patients who underwent surgical resection for HCC. The expression ratios of XIAP/XAF1 and of XIAP/Smac were also evaluated. Survivin, XIAP and XAF1 protein expression were investigated by immunohistochemistry. Correlations between mRNA levels, protein expression and clinicopathological features were assessed. Follow-up data were available for 69 HCC patients. The overall survival analysis was estimated according to the Kaplan-Meier method.

**Results:**

Survivin and Livin/ML-IAP mRNAs were significantly over-expressed in cancer tissues compared to non-neoplastic counterparts. Although Survivin immunoreactivity did not correlate with qPCR data, a significant relation was found between higher Survivin mRNA level and tumor stage, tumor grade and vascular invasion.

The mRNA ratio XIAP/XAF1 was significantly higher in HCCs than in cirrhotic tissues. Moreover, high XIAP/XAF1 ratio was an indicator of poor prognosis when overall survival was estimated and elevated XIAP immunoreactivity was significantly associated with shorter survival.

**Conclusion:**

Our study demonstrates that alterations in the expression of IAP family members, including Survivin and Livin/ML-IAP, are frequent in HCCs. Of interest, we could determine that an imbalance in XIAP/XAF1 mRNA expression levels correlated to overall patient survival, and that high XIAP immunoreactivity was a poor prognostic factor.

## Background

Hepatocellular carcinoma (HCC), one of the most common malignant tumors worldwide, can be managed with surgical resection or transplantation in selected cases, whereas advanced tumors responds poorly to currently available medical therapies [1].

The understanding of the molecular pathways leading to the development of HCC may provide important data to develop new therapies. Similarly to other tumor types, an imbalance between unrestrained cell proliferation and impaired apoptosis appears to be a major unfavorable feature of HCC [2]. Recent studies have documented the over-expression of anti-apoptotic factors such as the inhibitors of apoptosis proteins (IAPs) in a variety of solid tumors and cancer cell lines [3].

Eight human IAPs have been identified so far: NAIP (BIRC1), c-IAP1 (BIRC2), c-IAP2 (BIRC3), X-linked IAP (XIAP, BIRC4), Survivin (BIRC5), Apollon (BRUCE, BIRC6), Livin/ML-IAP (BIRC7) and IAP-like protein 2 (BIRC8) [4]. The members of IAP family, defined by the presence of a baculovirus IAP repeat (BIR) protein domain, are key regulators of apoptosis, cytokinesis and signal transduction [3].

In addition to BIR domains, some members of this family as XIAP, c-IAP1, c-IAP2 and Livin/ML-IAP also have a RING domain that allows these proteins to act as E3 ubiquitin ligases [5]. The E3 ubiquitin ligase activity of the IAPs is capable of promoting ubiquitination and proteasomal degradation of caspases, TRAF2 and several other partners [4].

XIAP is unique among IAP proteins, because of its ability to inhibit and directly bind to activated caspases. Through its BIR2 domain with its N-terminal linker, XIAP binds to the active site of effectors caspase-3 or -7 and prevents substrate binding and subsequent catalysis [5]. On the other hand the BIR3 domain sequestrates active caspase-9. Furthermore, XIAP has been shown to promote nuclear factor -B (NF-B) activation by enhancing the translocation of NF-B from the cytoplasm into the nucleus, by increasing the degradation of inhibitor B protein and through its association with TAK1 kinase and its cofactor TAB1 [6].

The two main antagonists of IAP proteins are Smac/DIABLO and XAF1, involved in the balance and regulation of apoptotic stimuli. Smac/DIABLO is released from mitochondria together with cytochrome *c *after initiation of intrinsic apoptotic cascade. Smac appears to function as a general IAP inhibitor in that it is shown to bind to XIAP, cIAP1, cIAP2, Survivin, Livin/ML-IAP and BRUCE [3]. Conversely, the nuclear protein XAF1 exclusively interacts with XIAP, restraining this IAP anti-apoptotic action even in healthy cells. The mechanism by which XAF1 antagonizes XIAP is not completely explained, although recent studies showed that XAF1 is able to sequestrate XIAP from the cytoplasm into the nucleus. Moreover, XAF1 expression is low or absent in several tumor cell lines [7].

Since XIAP, Smac/DIABLO and XAF1 are antagonistic regulators, it is reasonable to assume that their relative expression ratios, rather than the expression of a single regulator, determine susceptibility for apoptosis [8].

This study aimed to investigate the gene and protein expression pattern of IAP family members and its antagonists in a series of human HCCs and to assess their clinical and prognostic significance.

## Methods

### Patients

Tissue samples were collected from 80 patients with liver cirrhosis who underwent surgical resection for HCC, between 1997 and 2007, in two hospitals (Istituto Clinico Humanitas and San Paolo Hospital, Milan). The study was carried out with Local Ethical committee approval. Patients included 62 men and 18 women (mean age: 67 years, range: 42–83). The number of patients positive for HCV, HBV was 54 and 9, respectively. None of the patients received chemotherapy, radiation and/or alcoholization therapy before surgery. In each case, samples of HCC and surrounding cirrhotic tissue were snap-frozen into cryovial with 1 ml of RNAlater^® ^and stored at -80°C. Routinely Hematoxylin-Eosin-stained sections of HCCs and surrounding tissue were reviewed by two pathologists according to WHO (2000) and TNM (2002). The HCCs were histologically graded as I-IV on the basis of cellular atypia and architectural complexity, according to Edmonson. The evaluated clinicopathological features were: sex, age at diagnosis, viral infection, tumour histological features (histotype, stage, number and diameter of nodules, presence of capsule, microvascular invasion, Edmonson's grade of differentiation). These are summarized in table 1.

**Table 1:** Patients' information.

Patient's age	≤67 y: 34	>67 y: 46		
Patient's sex	male: 62	female: 18		
Histotype	mixed: 24	trabecular: 51	pseudoglandular: 5	
Viral infection	HCV: 54	HBV: 9	None: 17	
Stage	pT1: 36	pT2: 33	pT3: 9	pT4: 2
Edmonson grade	I: 8	II: 35	III: 32	IV: 5
Capsule	NO: 34	YES: 46		
Vascular invasion	NO: 58	YES: 22		
N° nodule	1: 48	>1: 32		
Nodule size (cm)	≤3: 38	>3: 42		

Clinical outcome data were available in 69 patients (86%). The follow-up period ranged from 1 to 75 months (average 34 months). At the last follow-up, 15 patients were deceased for HCC disease, whereas 22 patients were alive with HCC recurrence.

### RNA extraction and Reverse Transcription

Samples were homogenized in 1 ml of TRIzol^® ^reagent (Invitrogen, Milan, Italy) with a tissue lyser (Qiagen). Total RNA was purified according to the manufacturers' protocol. Spectrophotometrical RNA quantification was conducted by GeneQuant II (Pharmacia Biotech) at 260 nm. Total RNA was stored at -80°C until molecular investigation was performed. In reverse transcription reactions, 1 μg of total RNA from each sample was used for cDNA generation in a final reaction volume of 100 μl with High Capacity cDNA Archive Kit (Applied Biosystems).

### Real Time RT-PCR

qPCR reactions of target (n = 9) and housekeeping (HKG n = 8) genes were performed using Assay-on-Demand™ chemistry in an ABI PRISM 7900 HT Sequence Detection System (Applied Biosystems, Foster City, CA, USA). The assay identification numbers of target and housekeeping genes are as follow: Hs00244967_m1 (BIRC1), Hs00357350_m1 (BIRC2), Hs00154109_m1 (BIRC3), Hs00236913_m1 (XIAP), Hs00213882_m1 (XAF1), Hs00153353_m1 (BIRC5), Hs00212288_m1 (BIRC6), Hs00223384_m1 (BIRC7), Hs00219876_m1 (Smac/DIABLO), Hs00609297_m1 (HMBS), Hs99999905_m1 (GADPH), Hs 99999907_m1 (β-2M), Hs00188166_m1 (SDHA), Hs00824723_m1 (UBC), Hs00427620_m1 (TBP), Hs99999909_m1 (HPRT1), Hs99999903_m1 (ACTβ). The BIRC5 assay is specific for its two splicing variants: NM_001168.2 and NM_001012271.1 (Survivin and Survivin2B, respectively). The BIRC7 assay recognizes all splicing variants of this gene.

Instrument raw data (fluorescence) of all the samples were converted in threshold cycles (Ct) by SDS 1.2 software (Applied Biosystem, Foster City, CA, USA). Ct values were then imported in Excel worksheet for relative quantification (RQ).

For RQ calculation, the geometrical mean of the three more stable HKGs (HMBS, β-2M and GAPDH, GeNorm software) [9], was used as normalization factor.

The Fold Change (FC) was calculated, defined as the ratio between averaged normalized expression level of targets in neoplastic and corresponding non-neoplastic samples. Normalized RQ were log2-transformed for statistical analysis.

The ratios of XIAP and its antagonists were calculated as follows: first, for every patient, the ratio between normalized expression level of XIAP and its antagonists both in cancer and in cirrhotic tissue was calculated. Then the average value of these single ratios for all cancerous and cirrhotic samples, respectively, was calculated. Finally the fold change ratios of XIAP/XAF1 and XIAP/Smac were calculated.

### Tissue Microarray (TMA) construction

Representative tissue blocks from surgical resection of 40 patients (all included in qPCR study) were chosen to construct 2 TMA as previously described [10]. For each patient, seven cylindrical tissue cores were included in the TMA: three from carcinoma, two from cirrhotic tissue close to the cancer (< 1 cm) and two cirrhotic tissues taken at a distance of at least 3 cm from neoplastic lesion. From each TMA block a 4 μm-thick section was cut, stained with H&E and inspected for adequacy before immunohistochemical studies.

### Immunohistochemistry

Serial 4 μm-thick sections from each TMA block were stained with a series of primary antibody: XIAP (clone 48, BD Biosciences, San Jose, CA USA) 1:100; XAF1 (ab32023, Abcam, Cambridge, USA) 1:100; full-length Survivin (NB 500-201, Novus Biologicals, Littleton, USA) 1:2000. Immunohistochemistry was performed using a Dako immunostainer (Dako, Glostrup, Denmark) and immunostaining was revealed by Dako EnVision™ Detection Kit with Peroxidase/DAB as chromogen. All slides were counterstained with haematoxylin.

Immunohistochemical results were evaluated by two pathologists blinded to clinical data. Intensity and percentage of positive cells were calculated by averaging out replicate cores. The percentage of immunoreactive tumor cells and cirrhotic liver cells was determined and assigned to one of the following five categories: 0, (<5%); 1, (5%–24%); 2, (25%–49%); 3, (50%–74%) and 4, (≥75%). The intensity of immunostaining was scored as absent (0), mild (1), moderate (2) and marked (3). The percentage of positive cells and staining intensity were multiplied to produce a weighted score for each case. Cases with weighted score 0 were defined as negative; 1–6 as low expressors and 7–12 as high expressors.

### Statistical analysis

Statistical analysis was conducted for both sets of results: those from qPCR and those relative to immunohistochemical studies. For samples clustering (qPCR results), log2-transformed data were imported in dChip software (dChip 2006, DNA-Chip Analyzer available for free at http://www.hsph.harvard.edu/~cli/complab/dchip). Relative abundance of each gene was standardized by software pre-processing steps (subtracting its mean and dividing the result by its standard deviation) and hierarchical clustering analysis was then performed. The 1-Rank correlation was employed as distance metric and the Average as a linkage method.

The genes with a global FC exceeding 2 or lower than 0.5 were statistically analyzed by univariate statistics (paired t test).

For statistical purposes tumors with Edmondson's grade I and II, III and IV were categorized in "low" and "high" grade subsets, respectively. HCCs staged pT3 and pT4 were merged in the same group. Correlations of gene and protein expression with clinicopathological features (histotype, viral infection, stage, grade, vascular invasion, perineoplastic capsule, number of nodules, and diameter of nodules) were analyzed by ANOVA and by Chi-square, respectively.

To investigate whether expression levels of each gene and protein were associated to patient survival, HCC samples were categorized in high or low expressor groups if the target level was above or below the median expression value, respectively. For XIAP/XAF1 and XIAP/Smac mRNA ratios correlation to patients' outcome, HCCs were categorized in "low" and "high" groups if the ratio value was lower or higher than 2, respectively.

Overall and disease-free survival curves of HCC patients were plotted according to the Kaplan-Meier method using the log-rank statistics to test for statistical significant difference of generated curves (GraphPad Prism version 4.00 for Windows, San Diego, California, USA, http://www.graphpad.com). Data with a p value lower than a threshold limit (p < 0.05) were considered significant.

## Results

### Unsupervised analysis of IAP family members and their antagonists distinguishes HCC from non-neoplastic samples

All investigated genes were expressed at detectable levels (Ct <40) in all HCCs and corresponding non-neoplastic samples. To explore whether HCC and non-neoplastic tissue could be distinguished at molecular level by the expression profile of IAPs and inhibitors, unsupervised hierachical clustering (dChip) was performed. The majority of HCC samples (K, n = 62, 88%, p < 0.0001 by Fisher's exact test) clustered in the same branch of the dendrogram clearly separated from matched non-neoplastic counterparts (N) (see Additional file 1).

### Expression of IAPs and its antagonists in HCC

Survivin and Livin/ML-IAP mRNAs exhibited significant overexpression in cancer tissue compared to non-neoplastic tissue (FC = 6.86, p < 0.001 and FC = 2.33, p < 0.001, respectively) (table 2). Only NAIP mRNA revealed a slightly lower expression level in cancer tissue compared to non-neoplastic tissue, although the fold change was not significant (FC = 0.67). The expression of other genes was comparable between cancer and non-neoplastic tissue (FC between 0,91 and 1.15).

**Table 2:** IAPs, XAF1, Smac/DIABLO normalized mRNA level and ratios.

	Non neoplasia	Hepatocarcinoma	Fold Change	p value*
NAIP (BIRC1)	1,24 (0,01–2,6)	0,82 (0,0004–3,6)	0,67	
c-IAP1 (BIRC2)	2,13 (0.06–5.5)	1,94 (0.01–5.8)	0,91	
c-IAP2 (BIRC3)	1,23 (0.1–6.1)	1,21 (0.04–5.9)	0,99	
XIAP (BIRC4)	0,86 (0.3–2.5)	0,99 (0.06–2.2)	1,15	
**Survivin (BIRC5)**	**0,03 (0.03–0.1)**	**0,20 (0.1–1.6)**	**6,86**	**1·10–6**
Bruce (BIRC6)	1,36 (0.06–5.9)	1,36 (0.02–3.3)	1,00	
**Livin/ML-IAP (BIRC7)**	**0,13 (0.01–1.5)**	**0,31 (0.01–3.6)**	**2,33**	**6·10–3**
XAF1 (BIRC4BP)	0,69 (0.1–2.4)	0,61 (0.07–3.9)	0,88	
Smac/DIABLO	1,03 (0.4–2.1)	1,13 (0.4–3.6)	1,10	
**Ratio XIAP/XAF1**	**2,29 (0.3–12.9)**	**6,90 (0.2–47.0)**	**3,02**	**3·10–4**
Ratio XIAP/Smac/DIABLO	0,92 (0.04–2.0)	1,00 (0.01–4.2)	1,09	

* Student t-test.

Expression values of XAF1 and Smac/DIABLO were comparable between HCCs and non-neoplastic tissues (FC = 0.88 and FC = 1.10 respectively).

XIAP/XAF1 ratio was significantly higher in tumor than in non-neoplastic parenchyma (FC = 3.02, p < 0.001; table 2). On the contrary Smac/DIABLO expression did not show any significant difference between HCC and non-neoplastic liver (FC = 1.09).

### IAPs gene expression and clinical pathological features

A significant correlation was found between high Survivin mRNA level and high tumor stage (pT3 and pT4, p = 0.03), high tumor grade (III and IV, p = 0.01) and vascular invasion (p = 0.001). Moreover, high c-IAP2 mRNA level was found to be significantly correlated with absence of perineoplastic capsule (p = 0.02) and high NAIP mRNA level with pseudoglandular histotype (p = 0.03). Finally, high mRNA levels of c-IAP1 and Smac/DIABLO genes were significantly correlated with younger patient age (p = 0.03 and p = 0.009 respectively).

### IAPs gene expression and prognosis

**Figure 1:**
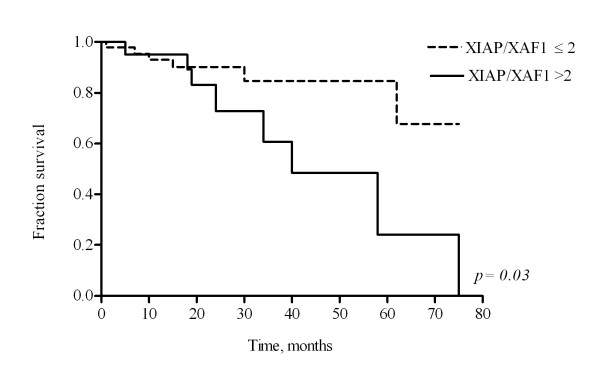
**High XIAP/XAF1 ratio is a poor prognostic factor in HCC patients**. Kaplan-Meier survival curves of HCC patients characterized by XIAP/XAF1 mRNA ratio > 2 (high, solid line) or ≤ 2 (low, dashed line) when compared by log-rank test. Overall survival was significantly different among the two subgroups of patients (p = 0.03).

Livin/ML-IAP and Survivin overexpression showed no correlation with disease outcome, although there was a trend for shorter overall survival in patients with high Survivin expression (p = 0.09). Kaplan-Meyer analysis demonstrated a significantly shorter overall survival in patients with high XIAP/XAF1 ratio (p = 0.03) (figure 1).

### IAPs immunoreactivity and prognosis

The gene expression profiles obtained suggested that Survivin, XIAP and XAF1 could be important in determining clinical outcomes of HCC patients. To confirm this hypothesis, we investigated Survivin, XIAP and XAF1 protein expression by immunohistochemistry on a tissue microarray platform.

**Figure 2:**
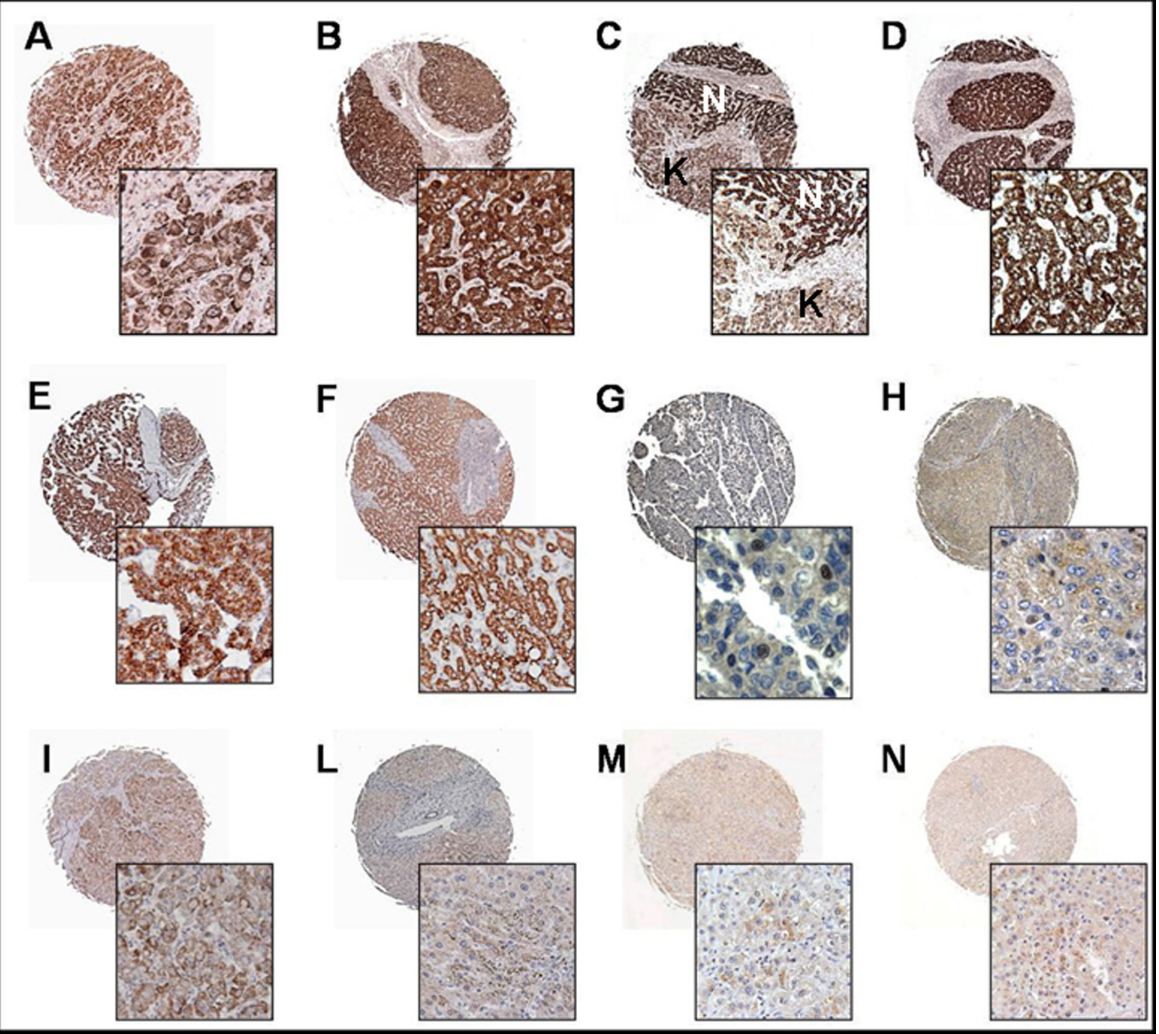
**Protein expression pattern of Survivin, XIAP and XAF1 in HCC tissues and non-neoplastic liver parenchyma**. IAP members immunoreactivity was estimated by tissue microarray in a subset of HCC patients (n = 40). A-D, Representative Survivin cytoplasmatic immunostaining in a tumor core (A), in a tumor proximal to cirrhosis (C, N: cirrhosis, K: HCC), and in adjacent and long-distance non-neoplastic parenchyma (B and D, respectively). XIAP marked (score 12) and moderate (score 8) immunoreactivity is shown for HCC (E and I, respectively) as well as for cirrhosis (F and L, respectively). G and H, Nuclear XAF1 staining is shown for tumor and non-neoplastic liver whereas XAF1 cytoplasmatic expression in HCC and cirrhosis is shown in panels M and N, respectively. Original magnification ×50 and ×250, for tissue cores and insets, respectively.

Survivin immunoreactivity was intense in the cytoplasm of cirrhotic liver cells, whereas HCC cells generally showed weak cytoplasmic staining (figure 2). Nuclear Survivin immunoreactivity was found only in five HCC (12,5%). Survivin immunoreactivity was not associated with any clinical variable: although samples of HCC with high level of Survivin were correlated with a shorter overall survival, this trend did not reach statistical significance (p = 0.39).

**Figure 3:**
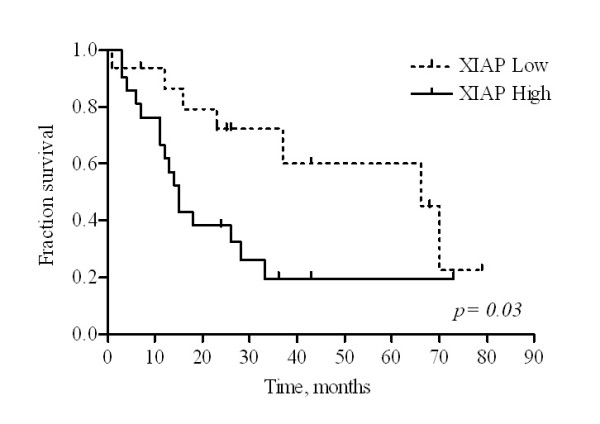
**High XIAP expression is a poor prognostic factor in HCC patients**. Kaplan-Meier survival curves of HCC patients characterized by high XIAP expression (solid line) or low (dashed line) when compared by log-rank test. Overall survival was significantly different among the two subgroups of patients (p = 0.03).

XIAP immunoreactivity was detected exclusively in cytoplasm of both neoplastic and non-neoplastic liver cells. Most HCC (82,5%, n = 33) displayed XIAP immunoreactivity, whereas 70% (n = 28) of cirrhotic liver tissue (distant from the tumor) expressed XIAP (table 3). Survival analysis demonstrated a significantly shorter overall survival in patients with high XIAP expression (p = 0.03) (figure 3); no correlations with other clinicopathological parameters were found.

**Table 3:** Survivin, XIAP and XAF1 immunoreactivity.

		Survivin		XIAP		XAF1	
	IHC score*	**n**	**(%)**	**n**	**(%)**	**n**	**(%)**

HCC	Negative	-		7	17,5%	23	57,5%
	Low	22	55,0%	23	57,5%	17	42,5%
	High	18	45,0%	10	25,0%	-	
Adjacent liver^#^	Negative	-		10	25,0%	21	52,5%
	Low	2	5,0%	19	47,5%	19	47,5%
	High	38	95,0%	11	27,5%	-	
Non-adjacent liver^#^	Negative	-		12	30,0%	25	62,5%
	Low	1	2,5%	18	45,0%	15	37,5%
	High	39	97,5%	10	25,0%	-	

The number (n) and the percentage of patients (%) in each IHC category are indicated.

*Negative (score 0), Low (score 1–6) and High (score 7–12).

#Adjacent liver: liver parenchyma at less than 1 cm from the tumor; non-adjacent liver: liver parenchyma at more than 3 cm from tumor.

XAF1 immunoreactivity was detected in the cytoplasm and nucleus of neoplastic and cirrhotic liver cells (figure 3). More than fifty percent of the HCC and cirrhotic tissues did not show XAF1 expression (table 3) and the expression score of XAF1 was significantly lower than that of XIAP. XAF1 immunoreactivity did not reveal any significant correlation with clinicopathological parameters and survival.

Since qPCR results showed that XIAP/XAF1 ratio strongly correlated with prognosis, we divided patients in four groups for survival analysis, depending on the following immunoreactivity patterns: group 1, XIAP low and XAF1 negative; group 2, XIAP low and XAF1 positive; group 3, XIAP high and XAF1 negative and group 4, XIAP high and XAF1 positive. Interestingly, groups 3 and 4 showed a trend for worse disease free and overall survival, although it did not reach statistical significance (p = 0,10 and p = 0,08 respectively) (data not shown).

## Discussion

This study is the first comprehensive evaluation of the gene expression of all IAP family members in a large number of human HCCs and paired cirrhotic parenchyma.

Our group and others have previously reported that Survivin is overexpressed in HCC [11,12]. Because of the significant homology among IAP genes, it has been hypothesized that other IAPs in addition to Survivin could be important in HCC pathobiology.

The results of this study show that both Survivin and Livin/ML-IAP mRNAs are overexpressed in HCC, although the expression levels of these two genes are not significantly associated with patient's survival. Survivin overexpression is related with clinicopathological factors, as high tumor grade, vascular invasion and higher tumor stage (pT3 and pT4) in accordance with previous literature [11,12]. However, the immunohistochemical analysis showed a reduced Survivin expression in HCC compared with the paired cirrhotic tissue and only a minority of HCC cores displayed nuclear Survivin immunoreactivity. This observation confirms the data reported by Chau *et al. *[13]. In addition these authors confirmed by western blotting analysis that Survivin protein was more abundant in non-tumor liver tissues than in HCC samples [13].

Takashima *et al. *reported that the level of Survivin mRNA increases along with the progression of chronic liver injury, suggesting that Survivin may be an essential factor in the survival of hepatocytes [14]. Recently, p53 dysfunction and hepatitis B virus infection have been shown to regulate the expression of Survivin in HCC cells [15].

The prognostic role of Survivin in HCC is still unclear: some studies support its correlation with poor prognosis whereas other reports do not [16,17]. This discrepancy may be explained with the multifunctional role of Survivin, as cell cycle and apoptosis-related protein [18]. Moreover, in response to cell-death stimuli, Survivin directly interacts with XIAP by conserved BIR domain, increasing XIAP stability, and indirectly sequesters Smac/DIABLO away from XIAP [19].

Importantly, four Survivin isoforms (Survivin, Survivin-2B, Survivin-ΔEx3 and Survivin-3B) have been described. Recent transfection experiments documented different roles for Survivin isoforms: Survivin-ΔEx3 retains the same anti-apoptotic properties of Survivin, whereas Survivin-2B shows markedly reduced anti-apoptotic properties. Moreover different Survivin isoforms show opposite roles in disease relapse and tumor cell survival in non-small-cell lung cancer (NSCLC) [20]. Post-transcriptional regulatory mechanisms have been described and may play a role in regulating Survivin expression, causing discrepancies between Survivin mRNA and Survivin immunoreactivity. Indeed these mechanisms include rapid changes in Survivin protein stability modulated by phosphorylation [21], subcellular trafficking controlled by monoubiquitination [22] and dynamic exchange of Survivin pools among individual subcellular compartments [23].

Livin/ML-IAP mRNA has been found overexpressed in some tumors including melanoma, breast, cervical, colon and prostate cancers, as well in leukemia, in lymphoma and in hepatoma cell lines. It has been proposed that endogenous Livin/ML-IAP has a minor direct effect on caspase activity whereas its anti-apoptotic effect could be ascribed to its antagonizing activity on the XIAP-Smac/DIABLO interaction. Moreover, researchers have shown that only overexpression of Livin-α isoform is correlated with high risk of relapse in bladder cancer [24]. To better understand the role of Livin/ML-IAP in liver carcinogenesis, future research needs to investigate the expression and localization of the protein and on the relationship between Livin/ML-IAP α and β isoforms in HCC.

In this study we observed NAIP mRNA to be under expressed in HCC compared to non-neoplastic liver parenchyma, though not at a significant level. It must be noted, however, that NAIP is highly expressed in macrophages [25] and therefore its expression by Kupffer cells, which are abundant in non neoplastic liver tissue, may account for this result.

Although overexpression of c-IAP1, c-IAP2 in renal cell carcinoma and in hepatoma cell line expressing hepatitis B virus has been reported [26,27], and Apollon upregulation was associated to chemoresistance *in vitro *and with unfavorable clinical features at diagnosis [28,29], our results showed that the gene expression levels of these IAP members were similar in HCC and liver parenchyma.

Increased XIAP has been reported in a variety of human tumors, including oesophageal carcinoma, clear cell renal carcinoma, ovarian carcinoma, and lymphoma [30]. In hepatocellular carcinoma XIAP overexpression compared to non-cancerous tissues is equivocal [31,32], though XIAP expression correlated with HCC recurrence and patient survival after treatment [30]. We could correlate high levels of XIAP immunoreactivity with decreased survival time in our series of HCC patients.

Apoptosis is controlled by the balance of antiapoptotic (as IAPs) and pro-apoptotic regulators, as XAF1 and Smac/DIABLO. These proteins are direct partners and inhibitors of XIAP [5]. The down regulation of XAF1 and/or Smac/DIABLO has been confirmed in a variety of cancer cells and tumor tissues [32,33]. The analyses conducted in this study show that the expression levels of XAF1 are similar in HCC and cirrhotic tissue, either in terms of mRNA expression or in terms of protein expression, as previously reported [31]. The mRNA levels of Smac/DIABLO did not varied significantly in our series of HCCs and matched non-neoplastic counterparts [34].

Recent observations indicate a tight regulation of all components and suggest that the relative expression ratio of these antagonistic regulators, rather than the expression of a single factor, determines susceptibility for apoptosis. Studies have reported that the balance between XIAP and Smac/DIABLO expression is gradually disturbed during progression of renal cell carcinomas and testicular germ cell tumors [19,35]. Similarly it was shown that the expression ratio of XIAP and XAF1 determines resistance or sensitivity of motoneurons to apoptosis [36]. Moreover, in gastric adenocarcinomas, the expression ratio between XIAP and XAF1 increases [8] and the expression balance of XIAP and XAF1 is an independent prognostic factor [37].

This study demonstrates that the expression ratio between XIAP and proapoptotic XAF1 is significantly higher in HCC, whereas the ratio between XIAP and Smac/DIABLO is comparable in neoplastic and non-neoplastic tissue. Moreover the imbalance between XIAP and XAF1 expression strongly correlate with poor prognosis. This result was confirmed when relative protein levels were considered, and patients with XIAP-positive/XAF1-negative expression pattern showed a trend towards worse overall and disease-free survival.

Therefore the imbalances between anti- and pro-apoptotic factors may result in an increased effect of XIAP, thereby generating an important selective survival advantage of HCC cells. The inappropriate increase of antiapoptotic XIAP over proapoptotic XAF1 in HCC may also contribute to the well-known clinical resistance to anticancer drugs.

## Conclusion

This study shows that alterations in the expression of IAP family members, as well as a marked imbalance of the expression ratio of antagonistic regulators, such as XIAP/XAF1, play an important role in HCC and patient's prognosis. Survivin and Livin/ML-IAP overexpression in HCCs imply that their mRNA levels could be used as markers of cancer tissue. XIAP expression levels (both mRNA and protein) did not show any significant difference in HCC tissue compared to cirrhotic tissue. Nonetheless its immunoreactivity correlated to patient's prognosis. More importantly, XIAP/XAF1 expression ratio could be related with patients' survival.

## Competing interests

The authors declare that they have no competing interests.

## Authors' contributions

CA and LC performed laboratory work, evaluated gene and protein expression, performed statistical analysis and wrote the manuscript. CP and VV performed laboratory work and helped with data analysis. MM, MR MMontorsi, GT, RS and MD collected and analyzed clinical and pathological data, including patients' follow-up. SB, GC and MR planned the investigation, supervised the laboratory work, and critically discussed data analysis. All the authors revised and approved the final version.

## Pre-publication history

The pre-publication history for this paper can be accessed here:

http://www.biomedcentral.com/1471-2407/9/125/prepub

## Supplementary Material

Additional file 1 **Unsupervised analysis of Inhibitor of Apoptosis Proteins in hepatocellular carcinoma tissues and non-neoplastic parenchyma**. HCC tissues (K, black color) could be clearly distinguished from non-neoplastic parenchyma (N, blue color) by hierarchical clustering analysis (p < 0.001, chi-square test). Click here for file
