# Supplementary figures and images for: Inhibitors of apoptosis proteins (IAPs) expression and their prognostic significance in hepatocellular carcinoma
Source: BMC Cancer. 2009 Apr 27;9:125. doi: 10.1186/1471-2407-9-125 (PMC2680906; doi:10.1186/1471-2407-9-125)

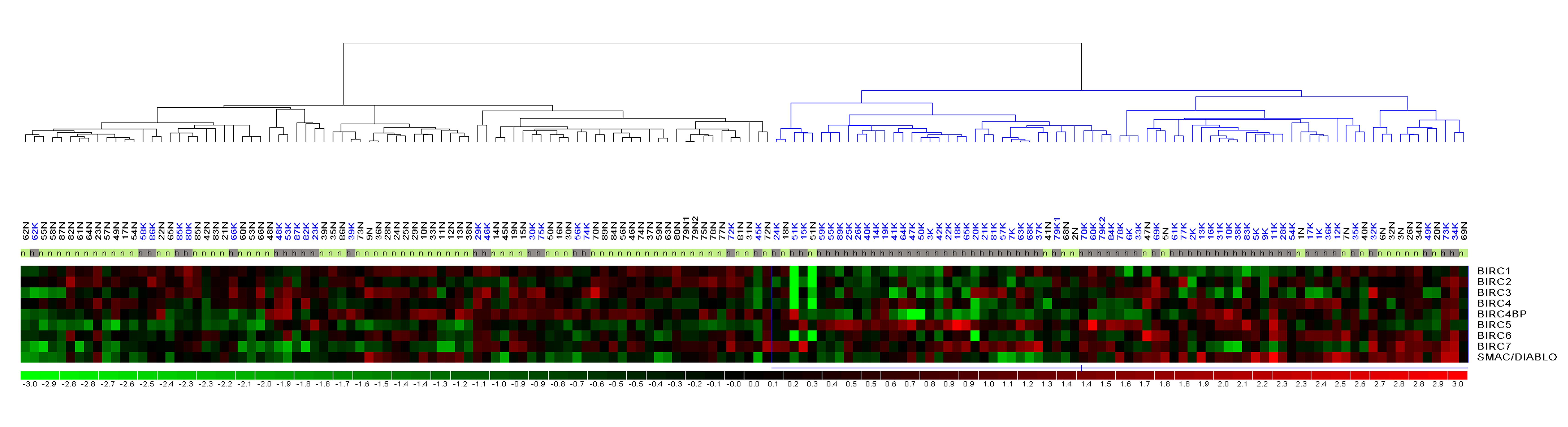

Supplement: Additional file 1 — Unsupervised analysis of Inhibitor of Apoptosis Proteins in hepatocellular carcinoma tissues and non-neoplastic parenchyma. HCC tissues (K, black color) could be clearly distinguished from non-neoplastic parenchyma (N, blue color) by hierarchical clustering analysis (p < 0.001, chi-square test). [file 1471-2407-9-125-S1.tiff]
